# Field validation of recombinant antigen immunoassays for diagnosis of Lassa fever

**DOI:** 10.1038/s41598-018-24246-w

**Published:** 2018-04-12

**Authors:** Matthew L. Boisen, Jessica N. Hartnett, Jeffrey G. Shaffer, Augustine Goba, Mambu Momoh, John Demby Sandi, Mohamed Fullah, Diana K. S. Nelson, Duane J. Bush, Megan M. Rowland, Megan L. Heinrich, Anatoliy P. Koval, Robert W. Cross, Kayla G. Barnes, Anna E. Lachenauer, Aaron E. Lin, Mahan Nekoui, Dylan Kotliar, Sarah M. Winnicki, Katherine J. Siddle, Michael Gbakie, Mbalu Fonnie, Veronica J. Koroma, Lansana Kanneh, Peter C. Kulakosky, Kathryn M. Hastie, Russell B. Wilson, Kristian G. Andersen, Onikepe O. Folarin, Christian T. Happi, Pardis C. Sabeti, Thomas W. Geisbert, Erica Ollmann Saphire, S. Humarr Khan, Donald S. Grant, John S. Schieffelin, Luis M. Branco, Robert F. Garry

**Affiliations:** 1 Zalgen Labs, LCC, Germantown, MD USA 20876 and Aurora, CO 80013 Germantown, USA; 2 Tulane University, Department of Microbiology and Immunology, New Orleans, LA 70112 USA; 3 Tulane University, School of Public Health and Tropical Medicine, New Orleans, LA 70112 USA; 4 Viral Hemorrhagic Fever Program, Kenema Government Hospital, Kenema, Sierra Leone; 5 Ministry of Health and Sanitation, Freetown, Sierra Leone; 6 Eastern Polytechnic Institute, Kenema, Sierra Leone; 7 University of Texas Medical Branch, Galveston National Laboratory, Galveston, TX USA; 8 Center for Systems Biology, Department of Organismic and Evolutionary Biology, Harvard University, Cambridge, Massachusetts, 02138 USA; 9 The Broad Institute of MIT and Harvard, Cambridge, Massachusetts, 02142 USA; 10 Department of Immunology and Infectious Diseases, Harvard T.H. Chan School of Public Health, Harvard University, Boston, Massachusetts, 02115 USA; 11 Autoimmune Technologies, LLC, New Orleans, LA USA; 12 Department of Immunology and Microbial Science, The Scripps Research Institute, La Jolla, California, 92037 USA; 13 Scripps Translational Science Institute, La Jolla, California, 92037 USA; 14 Department of Integrative Structural and Computational Biology, The Scripps Research Institute, La Jolla, California, 92037 USA; 15 Department of Biological Sciences, College of Natural Sciences, Redeemer’s University, Ede, Osun State Nigeria; 16 African Center of Excellence for genomics of Infectious Diseases, Redeemer’s University, Ede, Osun State Nigeria; 17 Howard Hughes Medical Institute, Chevy Chase, Maryland, 20815 USA; 18 The Skaggs Institute for Chemical Biology, The Scripps Research Institute, LaJolla, CA USA; 19 Tulane University, Department of Pediatrics, Section of Infectious Diseases, New Orleans, LA 70112 USA

## Abstract

Lassa fever, a hemorrhagic fever caused by Lassa virus (LASV), is endemic in West Africa. It is difficult to distinguish febrile illnesses that are common in West Africa from Lassa fever based solely on a patient’s clinical presentation. The field performance of recombinant antigen-based Lassa fever immunoassays was compared to that of quantitative polymerase chain assays (qPCRs) using samples from subjects meeting the case definition of Lassa fever presenting to Kenema Government Hospital in Sierra Leone. The recombinant Lassa virus (ReLASV) enzyme-linked immunosorbant assay (ELISA) for detection of viral antigen in blood performed with 95% sensitivity and 97% specificity using a diagnostic standard that combined results of the immunoassays and qPCR. The ReLASV rapid diagnostic test (RDT), a lateral flow immunoassay based on paired monoclonal antibodies to the Josiah strain of LASV (lineage IV), performed with 90% sensitivity and 100% specificity. ReLASV immunoassays performed better than the most robust qPCR currently available, which had 82% sensitivity and 95% specificity. The performance characteristics of recombinant antigen-based Lassa virus immunoassays indicate that they can aid in the diagnosis of LASV Infection and inform the clinical management of Lassa fever patients.

## Introduction

Lassa fever is a viral hemorrhagic fever (VHF) that is endemic in Sierra Leone, Guinea, Liberia and Nigeria, with cases reported in several other West African countries^1–5^. The etiologic agent of Lassa fever is Lassa virus (LASV; family *Arenaviridae*). LASV is transmitted to humans by contact with the excretions of its major reservoir *Mastomys natalensis*, an abundant peridomestic rodent, or during preparation of *Mastomys* as food^6,7^. After an incubation period of 7 to 21 days people infected with LASV can experience nondescript symptoms including fever, headache, malaise and general weakness^8–11^. An estimated 20% of individuals develop severe symptoms, including hypotension, neck and facial edema and vomiting after 4–7 days of mild illness^12^. Overt bleeding occurs in approximately 30% of severe cases and is predictive of an adverse outcome. In fatal cases, death due to multi-organ failure occurs between 10–14 days of symptom onset. The case fatality rate (CFR) was 69% in Lassa fever patients presenting while viremic to Kenema Government Hospital (KGH) in Sierra Leone between 2008–2012^1^. During a recent surge of Lassa fever cases in Nigeria the CFR among laboratory-diagnosed cases was 60%^13^. Women who are pregnant develop severe disease with increased frequency and have a Lassa fever CFR as high as 90%, with fetal death, miscarriage or spontaneous abortion occurring in nearly all cases^1,14,15^.

While there have been advances in understanding the molecular biology, immunology and genomics of LASV^2,16,17^, progress towards an immunotherapeutic drug^18^ and new initiatives to develop a Lassa fever vaccine^19^, at present there is no approved Lassa fever therapeutic or prophylactic. The nucleoside analog ribavirin is used off-label for treatment of Lassa fever, but must be administered early in infection to achieve modest efficacy^1,20–22^. Rapid diagnosis of LASV infection is imperative for proper patient management, which includes patient isolation, treatment with ribavirin when available, and supportive care such as the replacement of fluids and electrolytes. However, it is difficult to distinguish common febrile illnesses, such as malaria, typhoid fever, leptospirosis and arbovirus diseases, from Lassa fever on the basis of clinical presentation alone^23^. The ability to diagnosis Lassa fever is also critical because nosocomial infections can occur, especially in maternity or surgical wards where health care workers are exposed to blood and other bodily fluids^24^.

Laboratory diagnosis of Lassa fever is performed by isolating LASV in the blood or detecting LASV antigen (Ag), LASV genomic RNA, or LASV-specific antibodies in the blood^25–29^. While reverse transcriptase-polymerase chain reaction (RT-PCR) assays to detect LASV RNA can be used to diagnose Lassa fever^30–33^, the capacity to provide such testing as a routine method in rural public healthcare units (PHUs) in endemic regions of West Africa is limited at the present time. Immunoassays are used as the frontline diagnostic technique for many infectious diseases, including malaria, dengue, AIDS, rotavirus-induced diarrhea, and influenza^34^. Detection of proteins or antibodies is not susceptible to contamination issues that can produce false positive results in PCR-based assays^30^ or to the presence of PCR inhibitors that can produce false negative results^35^. The use of immunoassays based on broadly-reactive monoclonal or polyclonal antibodies can also potentially overcome sensitivity issues related to genetic diversity of RNA viruses such as LASV^27^. Moreover, rapid diagnostic tests, such as lateral flow immunoassays, can be effectively utilized in areas lacking advanced laboratory infrastructures and have a high rate of acceptance in these low resource settings^36^. Here, we compare the performance of Lassa fever immunodiagnostic assays based on recombinant Lassa virus (LASV) proteins, including a rapid diagnostic test (RDT), to available quantitative PCR (qPCR) assays.

## Results

### Recombinant LASV diagnostic assays

**Figure 1:**
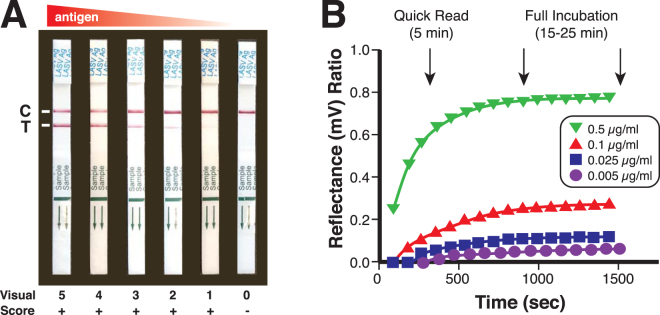
Development of a recombinant antigen LASV lateral flow immunoassay. (Panel A) The ReLASV RDT is designed as a dipstick style lateral flow immunoassay. It can be visually scored on a scale of 0 to 5. (Panel B) Scanning densitometry can quantify ReLASV RDT signal kinetics (mean of 3 scans at 90 sec intervals, ratio = test line ÷ control line). NP Ag concentrations are provided in the inset.

A suite of Lassa fever diagnostic immunoassays has been developed based on LASV recombinant proteins^1,23,29,37^. LASV IgM and IgG capture ELISAs utilize microwell plates coated with recombinant LASV (ReLASV) nucleoprotein (NP), glycoprotein complex (GPC), and Z matrix protein or with NP alone^37,38^. Plates in the current study were coated with LASV NP alone. The ReLASV NP has also been used as an immunogen to produce monoclonal antibodies (MAbs) in mice and polyclonal antibodies (PAbs) in animals of various species. These antibodies have been developed as LASV Ag-capture and detection reagents in ELISA format and a lateral flow immunoassay for use as a RDT. The ReLASV antigen-capture ELISA uses affinity-purified rabbit NP PAbs. The ReLASV RDT, a dipstick-style lateral flow immunoassay using paired LASV NP murine MAbs, is designed for detection of lineage IV LASV in blood (Fig. 1). The ReLASV can be visually scored on a scale of 0 to 5 (Fig. 1A), with strong positive samples developing in as little as 5 minutes with full signal development by 25 minutes (Fig. 1B).

**Figure 2:**
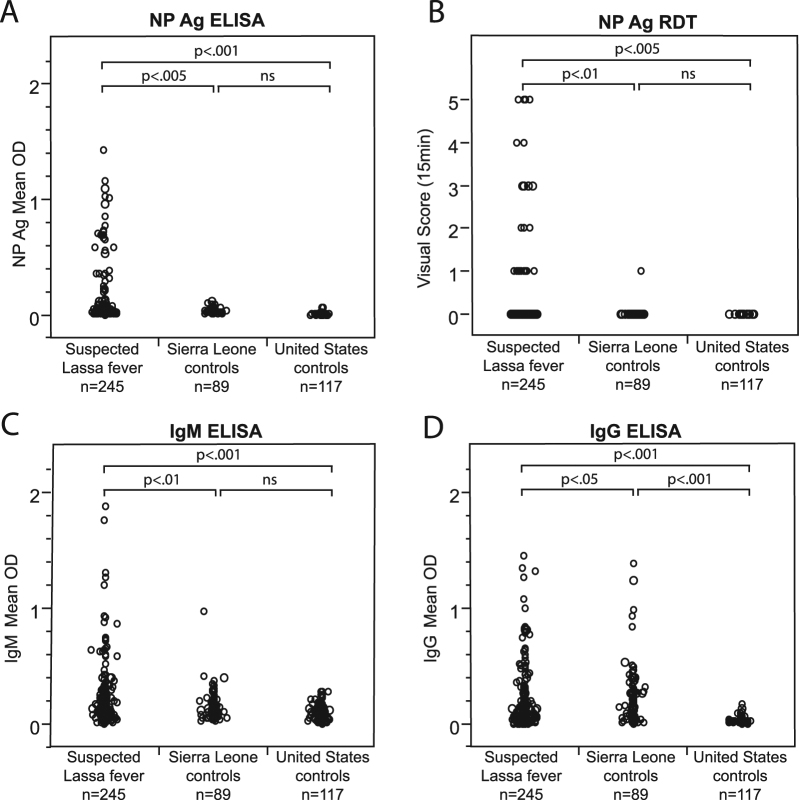
Clinical effectiveness of ReLASV RDT, and Ag, IgM, and IgG ELISAs. Comparisons between suspected Lassa fever cases and Sierra Leonean and United States control groups revealed significant differences in ELISA OD or mean visual score. (Panel A) Ag ELISA. (Panel B) RDT. (Panel C) IgM ELISA. (Panel D) IgG ELISA.

Kenema Government Hospital (KGH) is located in the Eastern Province of Sierra Leone, a region considered to have the highest per capita incidence of Lassa fever in the world^39^. A case definition based on major and minor clinical signs is used to identify suspected Lassa fever cases that present to the KGH VHF Ward (Table S1)^12^. We evaluated the field performance of the ReLASV immunoassays in serum and plasma samples from populations of individuals suspected of having Lassa fever and from control populations living in the Lassa fever endemic area of Sierra Leone or the United States (a non-endemic country) (Fig. 2). Both the ReLASV ELISA (Fig. 2A) and RDT (Fig. 2B) differentiated subjects meeting the Lassa fever case definition from Sierra Leonean or United States controls. This study also confirmed as expected that the detection of Lassa fever cases using measurement of either anti-LASV IgM (Fig. 2C) or IgG (Fig. 2D) is confounded by the LASV seroprevalence in the population within this LASV endemic region. Although a significant difference was noted between the anti-LASV IgM seroprevalence in suspected cases and Sierra Leonean controls, a fraction of currently healthy Sierra Leoneans had detectable anti-LASV IgM in their blood. While the presence of anti-LASV IgM antibodies can contribute to Lassa fever diagnosis, IgM persistence for months or longer in a subset of cases after resolution of symptoms confounds the reliability of IgM as an early marker for LASV infection^28,40^. Persistence of the anti-LASV IgM response has also been observed previously in a nonhuman primate model of Lassa fever^26^. Due to the endemic exposure of the Sierra Leonean population to LASV, the Sierra Leone control group registered significantly higher LASV IgG reactivity than the United States control group (p < 0.001).

### Comparison of ReLASV immunodiagnostics with qPCR

KGH and the Irrua Specialist Teaching Hospital (ISTH) located in Nigeria are the only two facilities in the world continuously testing for and admitting Lassa fever patients. The KGH laboratory under the guidance and oversight of Ministry of Health and Sanitation and the Pharmacy Board of Sierra Leone principally uses immunoassays, including the ReLASV ELISAs and RDT, for presumptive diagnosis of suspected Lassa fever cases^1,23,29,37^. Traditional RT-PCR based assays followed by detection of PCR products by agarose gel electrophoresis have been employed at KGH for research purposes, but were less reliable for patient screening due to false positive and false negative results. In contrast, ISTH has performed traditional PCR-based assays followed by detection of PCR products by gel electrophoresis for screening suspected Lassa fever cases for LASV infection^30^.

**Figure 3:**
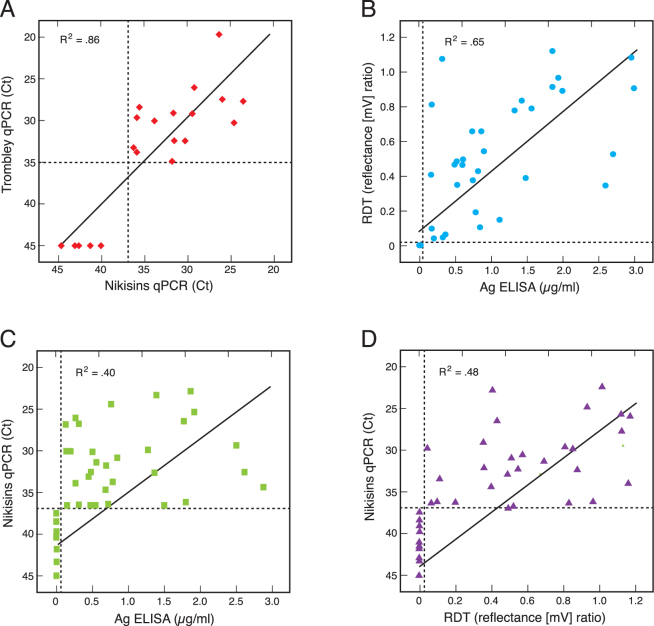
Correlations between quantitative values for quantitative polymerase chain reaction assays and ReLASV immunoassay. Cycle threshold (Ct) values, which are inversely related to viral genome equivalents, obtained by Nikisins or Trombley qPCR were compared (Panel A). Densitometry scan ratios between the test and control bands of ReLASV RDT and the amount of LASV antigen (µg/ml) detected by ReLASV (Panel B). Nikisins qPCR values were compared to the quantitative vales of the ReLASV ELISA (Panel C) or RDT (Panel C). Only concordant results were compared. R-squares (R^2^) values for fit to the linear regression line are indicated.

Quantitative PCR (qPCR) assays have been introduced by Trombley *et al*.^41^ and Nikisins *et al*.^33^. These qPCR assays and other qPCR assays in various stages of development are currently under evaluation at KGH and ISTH. To compare the performance of the ReLASV immunoassays to the Nikisins and Trombley qPCR assays we analyzed samples from suspected Lassa fever cases that presented to the KGH VHF Ward between January 2015 and August 2017. The study included 77 suspected Lassa fever cases that provided informed consent and whose samples had sufficient volume to run multiple diagnostic assays (Table S2). A Ct value of 37 was determined as the cut-off for Nikisins qPCR assay and a Ct value of 35 was determined as the cut-off for the Trombley qPCR assay using receiver operating characteristic (ROC) curves (Fig. S1, Table S3). Ct values above the cutoffs were considered negative. While the Nikisin qPCR assay detected a higher percentage of cases than the Trombley qPCR assay there was a high degree of correspondence (R^2^ = 0.86) between quantitative values (Ct scores) of the two qPCR assays when comparing concordant samples (Fig. 3A). When it became apparent that the Trombley qPCR assay was performing with reduced sensitivity compared to the Nikisins qPCR assay, only the Nikisins qPCR assay was employed in subsequent analyses to conserve the limited sample volumes available for this study and other research priorities at the KGH site. There was a moderate degree of correspondence (R^2^ = 0.65) between quantitative values (µg/ml Ag vs reflectance [mV] ratio) of the two immunoassays (Fig. 3B). Only a weak degree of correspondence was observed between the Ct values of the Nikisins qPCR assay and Ag levels detected by the ReLASV Ag ELISA (R^2^ = 0.40 Fig. 3C) and RDT (R^2^ = 0.48, Fig. 3D). The low correspondence of the qPCR assays and the Ag detection immunoassays suggests that LASV NP the may have different stability in blood than LASV RNA, or that it is released into blood in different amounts during the period when patients present for clinical evaluation.

### Lassa fever assay sensitivity and specificity

The ability to culture LASV from the blood of suspected cases at BSL-4 has been considered the gold standard to assess the performance of LASV diagnostic assays^27,42^. In the absence of BSL-4 virus culture capability Panning *et al*.^42^ suggested that the field performance of Lassa fever diagnostic assays could be evaluated using RT-PCR based assays and immunoassays. Using the Nikisins qPCR assay, the qPCR assay detecting the largest number of positive samples, as the diagnostic standard results in sensitivity of the ReLASV Ag ELISA of 94% and specificity of 84%; sensitivity of the ReLASV RDT is 91% and specificity is 86% (Table 1). Using the Nikisins qPCR assay as diagnostic standard results in a sensitivity of the Trombley qPCR assay of 76% and specificity of 100%.

**Table 1:** Performance of Lassa fever diagnostic assays using three different diagnostic standards.

Lassa fever diagnostic assay	Nikisins qPCR diagnostic standard	ReLASV Ag ELISA diagnostic standard	Combination diagnostic standard
**ReLASV Ag ELISA**			
Sensitivity	94.1% (78.9–99.0%)	—	95.0% (81.8–99.1%)
Specificity	83.7% (68.7–92.6%)	—	97.3% (84.2–99.9%)
PPV	82.1% (65.9–91.9%)	—	97.4% (84.9–99.9%)
NPV	94.7% (80.9–99.1%)	—	94.7% (80.9–99.1%)
Positive likelihood	5.8 (2.9–11.4)	—	35.2 (5.1–243)
**ReLASV RDT**			
Sensitivity	91.2% (75.2–97.7%)	94.8% (81.3–99.1%)	90.2% (75.9–96.8%)
Specificity	86.0% (71.4–94.2%)	100% (88.6–100%)	100% (88.9–100%)
PPV	83.7% (67.3–93.2%)	100% (88.3–100%)	100% (88.3–100%)
NPV	92.5% (78.5–98.05)	95.0% (81.8–99.1%)	90.0% (75.4–96.7%)
Positive likelihood	6.5 (3.9–13.8)	infinity	Infinity
**Nikisins qPCR**			
Sensitivity	—	82.1% (65.9–91.9%)	82.1% (65.9–91.9%)
Specificity	—	94.7% (80.9–99.1%)	94.7% (80.9–99.1%)
PPV	—	94.1% (78.9–99.0%)	94.1% (78.9–99.0%)
NPV	—	83.7% (68.7–92.75)	83.7% (68.7–92.7%)
Positive likelihood	—	15.6 (4.0–60.6)	15.6 (4.0–60.6)
**Trombley qPCR**			
Sensitivity	76.1% (52.3–90.1%)	60.7% (40.7–77.9%)	58.6% (39.1–75.9%)
Specificity	100% (80.0–100%)	100% (71.7–100%)	100% (73.2–100%)
PPV	100% (77.1–100%)	100% (77.1–100%)	100% (77.1–100%)
NPV	83.3% (61.8–94.5%)	54.2% (33.2–73.8%)	53.8% (33.7–72.9%)
Positive likelihood	infinity	infinity	Infinity

The use of qPCR assay that is susceptible to sequence variation of LASV as diagnostic standard can underestimate the specificity of the ReLASV immunoassays. Immunoassays are less susceptible to producing false negative results due to sequence variation than PCR-based assays. Using the ReLASV Ag ELISA, the assay detecting the largest number of positive samples, as the diagnostic standard results in sensitivity of the ReLASV RDT of 95% and specificity of 100% (Table 1). Under the Ag ELISA diagnostic standard, the Nikisins qPCR assays performs with a sensitivity and specificity of 82% and 95%, respectively; the sensitivity and specificity of the Trombley qPCR was 61% and 100%, respectively.

The performance of the ReLASV immunoassays was also compared to that of the LASV qPCRs using a combined diagnostic standard. In the combined algorithm performance of the ReLASV Ag ELISA, ReLASV RDT or qPCR assays was compared to results of the other assays. If the results of the ReLASV ELISA and RDT and the Nikisins qPCR were all positive, the case was considered a true positive. If the results were all negative, the case was considered a true negative. A case was considered a false positive or false negative if the results for an assay were discordant with the results of both of the other assays. A case was considered a false negative if another assay gave a positive result. Based on this combined standard, the sensitivity and specificity of the ReLASV ELISA was 95% and 97% respectively in this cohort of Sierra Leoneans with suspected Lassa fever (Table 1). The sensitivity and specificity of the ReLASV RDT was 90% and 100% respectively. Under the combined diagnostic standard the sensitivity of the Nikisins qPCR assay was 84% with 95% specificity and the sensitivity of the Trombley qPCR assay was 59% with 100% specificity.

**Figure 4:**
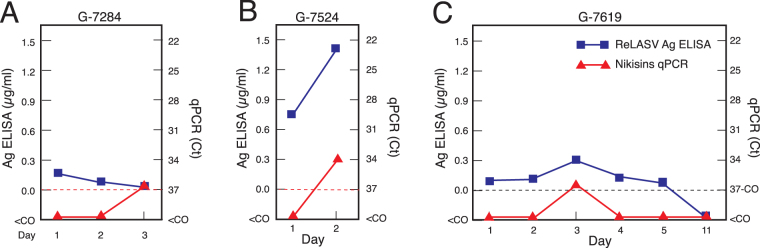
Earlier detection of Lassa virus in blood with ReLASV immunoassays than with quantitative polymerase chain reaction assays. Comparison of quantitative values for ReLASV Ag ELISA (µg/ml) and Nikisins qPCR (cycle threshold, Ct) in samples that were positive on ReLASV Ag ELISA and RDT on day of presentation (day 1), but negative on Nikisins qPCR are shown (panel A: G-7284, panel B: G-7524, panel C: G-7619). Blue squares: ReLASV Ag levels (µg/ml). Red triangles: Nikisins qPCR (Ct). Dashed line: Nikisins qPCR cycle threshold (Ct) cutoff = 37 cycles).

Multiple samples were available from a subset (30) of the 77 cases examined. Regardless of the diagnostic standard used (Nikisins qPCR, Ag ELISA or combined), a case was considered positive for an assay, if any sample from any day gave a positive result. In addition to eleven cases that were negative by one or both qPCR assays, but positive by both immunoassays, there were also several cases that were negative by both qPCR assays on presentation (day 1), but positive by the ReLASV ELISA (Fig. 4). Samples from cases G-7284 (Fig. 4A), G-7524 (Fig. 4B), and G-7619 (Fig. 4C) were positive by the Nikisins or Trombley qPCRs on blood draws taken at days 2 or 3 after presentation to KGH, confirming the positive results of the immunoassays. In a subset of patients LASV NP may appear earlier in the blood than LASV RNA.

**Figure 5:**
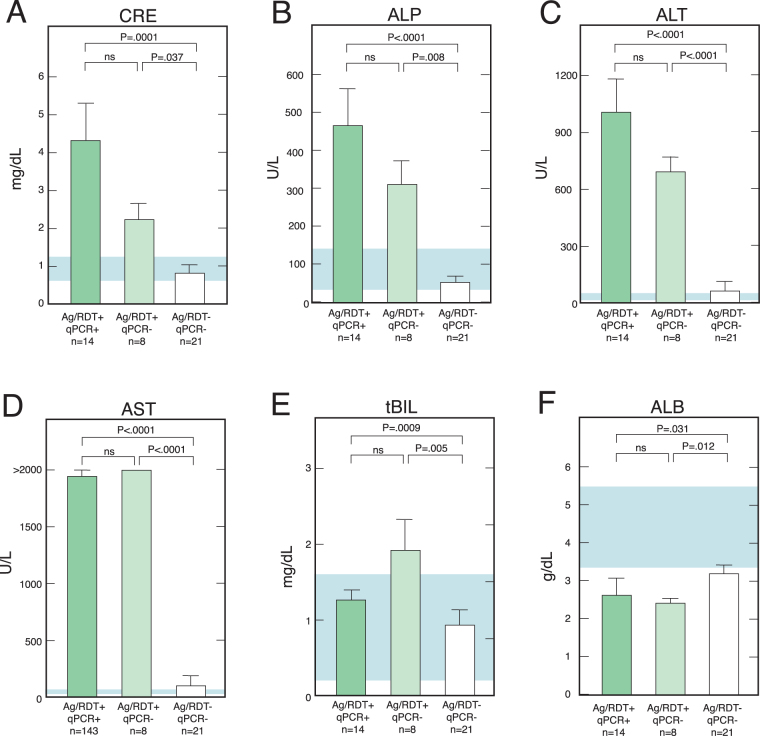
Clinical chemistry results for patients whose samples were antigen and RDT positive, but qPCR negative. Samples that were Antigen and RDT positive and negative on one or both qPCR assays with sufficient sample volume were compared using a Piccolo Clinical Chemistry Analyzer. Levels of blood creatinine (CRE), alkaline phosphatase (ALP), alanine transaminase (ALT), aspartate aminotransferase (AST). Total bilirubin (tBIL) and albumin are compared. Blue shading indicates normal ranges for the various measures. The T bars indicate standard errors.

The case fatality rate of cases with known outcomes that were considered positive by the Ag ELISA using the combined diagnostic standard was 85% (34/40) consistent with LASV infection. Samples that tested negative on one or both of the two qPCRs, but positive on the ReLASV immunoassays, also showed a high case fatality rate. Six of seven (86%) cases classified as false negative on the Nikisins qPCR assay by the combined diagnostic standard died, while 10/12 (83%) of cases classified as false negative on the Trombley qPCR assay died. Additionally, the qPCR negative/immunoassay positive cases had abnormal clinical chemistry profiles similar to those of subjects with positive qPCR results (Fig. 5). Subjects that were negative on qPCR assays, but positive on both ReLASV immunoassays had elevated blood levels of creatinine (CRE), alkaline phosphatase (ALP), alanine transaminase (ALT), aspartate aminotransferase (AST) and total bilirubin (tBIL), and decreased levels of albumin (ALB) comparable to cases that were qPCR positive. While a variety of conditions, including some infectious diseases, can result in abnormal clinical chemistry profiles, subjects that tested negative by ReLASV immunoassays but with altered clinical chemistry profiles presented infrequently in this cohort of suspected Lassa fever patients. With one exception among 21 tested, subjects that were negative by qPCR and immunoassays had clinical chemistry profiles that were not significantly different than healthy subjects.

### Inhibitors of qPCR reactions are not present in the blood of Lassa fever patients

Previous studies suggested that the blood of a subset of Lassa fever patients might contain inhibitors of PCR reactions^35^. Specimens at ISTH are tested by traditional PCR at both undiluted and 1:10 dilution^30^. The 1:10 dilution appears to reduce the concentration of the putative PCR inhibitor to below a concentration sufficient to interfere with the traditional RT-PCR assay used at ISTH. To determine whether or not the discrepant samples that were negative on qPCR assays, but positive on ReLASV immunoassays were negative because of the presence of a PCR inhibitor, we repeated a subset of the samples undiluted and after a 1:10 dilution (Supplementary Table 5). In this limited subset no undiluted samples that were negative on qPCR became positive after the 1:10 dilution.

### Performance of Lassa immunoassays and qPCR in Lassa fever contacts and controls

To further assess the performance of the ReLASV immunoassays, we tested serum from individuals who were asymptomatic, healthy family members or household contacts of Lassa fever patients. One of 25 contacts was positive by ReLASV immunoassays as well as both the Nikisins and Trombley qPCRs (Table S6). Contacts of Lassa fever patients that have LASV in the blood, but are not overtly ill, have been observed previously^29^ and are worthy of further study. Additionally, we tested non-African blood donors with seropositivity to other infectious agents including dengue virus, Epstein-Barr virus and parvovirus B19. None of these 44 samples test gave positive results on either the LASV qPCRs or immunoassays (Table S7). These results confirm that the LASV qPCRs and the ReLASV immunoassays have high specificity.

### Preliminary assessment of Pan Lassa RDTs

**Figure 6:**
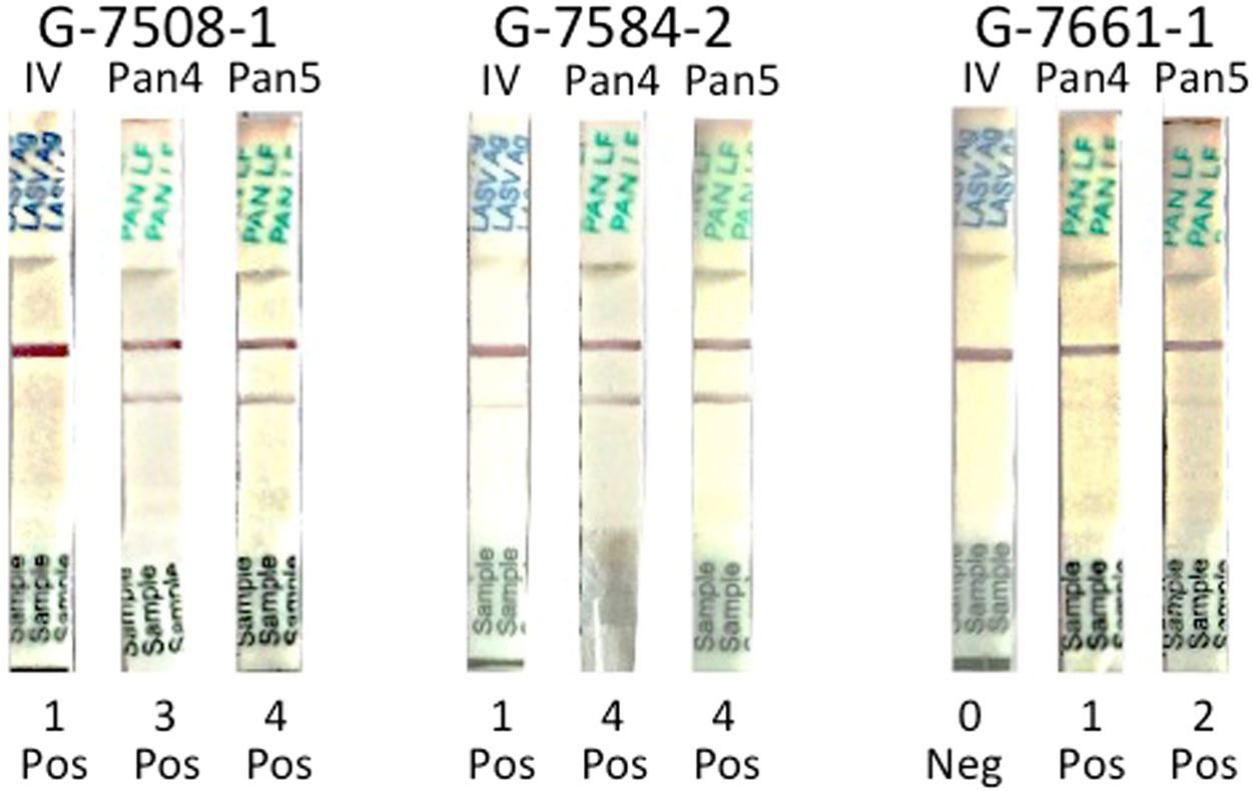
Increased sensitivity of prototype Pan-Lassa RDT over Josiah (lineage IV) RDT. Selected samples that were weak (G-7508-1, G-7584-2) or negative (G-7661-1) on the ReLASV lineage IV RDT were run on Pan Lassa RDT prototypes (Pan4 = lot 4, Pan 5 = lot 5).

The ReLASV RDT were designed for and validated against lineage IV LASV, which is present in the western part of the Lassa fever zone that includes Sierra Leone, Guinea, Liberia and surrounding countries^2,43^. The murine MAbs that serve as capture and detection reagents in the LASV lineage IV RDT perform with reduced sensitivity on Nigerian LASV at ISTH that are predominately lineage II. Development of Pan Lassa immunoassays that have a high sensitivity and specificity for detection of LASV from lineages present in Nigeria and other countries in the eastern side of the Lassa fever zone, while retaining reactivity as well to LASV linage IV is in process. Preliminary studies indicated that the Pan-Lassa RDTs, which are based on affinity purified rabbit PAbs, have improved sensitivity for diagnosis of both LASV lineage IV and Nigerian LASV infections. Two samples G-7508-1 and G-7584-2 that were weakly positive on LASV lineage IV RDT were more strongly reactive on Pan Lassa RDTs from two prototype lots (Fig. 6). A sample from G-7661 that was negative on the LASV lineage IV RDT Pan Lassa was reactive on Pan Lassa RDTs from two prototype lots (Fig. 6). A sample from G-7615 that was negative on the LASV lineage IV RDT Pan Lassa was shown to be reactive on a Pan Lassa RDT from an earlier prototype lot. These results suggest that Pan-Lassa RDTs based on polyclonal antibodies have comparable sensitivity to ReLASV ELISA, which contain similar polyclonal antibody reagents.

### Diagnostic algorithm

**Figure 7:**
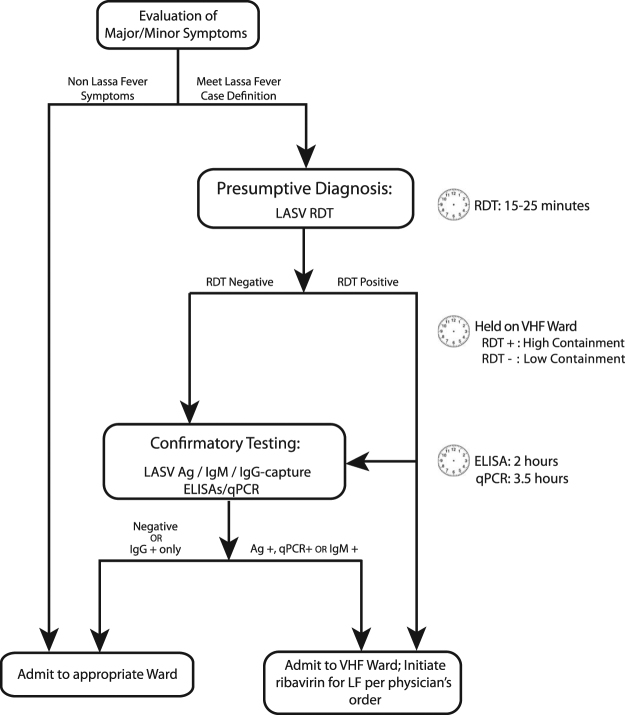
Proposed diagnostic algorithm for Lassa fever. Use of recombinant Lassa immunodiagnostics for presumptive diagnosis of patients with suspected Lassa fever at KGH and selected PHUs in Sierra Leone.

The use of the ReLASV RDT in combination with confirmatory testing by ReLASV ELISA and LASV PCR has enabled KGH to enhance its capacity to triage suspected cases of Lassa fever (Fig. 7). Upon arrival at KGH the patient is evaluated by a nurse or physician. If the patient meets the case definition of suspected Lassa fever^12^ based on signs and symptoms (Table S1), a blood specimen is tested on the LASV RDT, which provides a presumptive diagnosis. The attending physician may consider a positive RDT result as an aid to the diagnosis of Lassa fever, in addition to clinical signs and symptoms. Based on all these considerations, the patient may be moved to an isolation room on the VHF Ward and ribavirin treatment initiated per physician’s order. The suspected Lassa fever patient should remain in isolation even with a negative RDT, prior to subsequent testing by the ReLASV ELISAs and LASV PCR. Patients who test positive for Lassa fever by RDT or are positive by Ag-capture ELISA or IgM-capture ELISA may remain on the high containment ward for the course of ribavirin treatment and supportive care. Cases who test negative by RDT, but are positive by Ag-capture ELISA, are moved to the high containment ward for initiation of ribavirin and supportive care. Recombinant protein based diagnostics have helped redefine the diagnostic role of anti-LASV immunoglobulins^28^. If the patient is positive by IgM-capture ELISA, the physician will typically continue to monitor viral and antibody levels and treat at their discretion. Cases who present with a febrile illness, but no detectable LASV or antibody response are tested for Ebola virus (EBOV). Those who are positive by LASV IgG-capture ELISA only are transferred to an appropriate ward, depending on their signs and symptoms.

## Discussion

Infrastructure for performing PCR-based assays is limited in most Lassa fever endemic areas. There are also challenges for Lassa fever diagnosis by either nucleic acid or antigen detection assays because of high LASV genetic diversity^2^. Failure to amplify divergent LASV strains has been observed in previous studies of RT-PCR based LASV diagnostics^31,42,44^. For example, Trappier *et al*.^44^ identified only 82% of LASV culture positive cases by RT-PCR with amplicon detection by hybridization to a labeled probe. In this study the RT-PCR assay was positive on several samples that were virus culture negative indicating detection of LASV RNA associated with inactive or degraded virions, amplicon contamination or both. Our previous experience with the traditional LASV RT-PCR assays at KGH confirmed that they are susceptible to generating both false negative and false positive results, which prompted the development of ReLASV immunoassays, as well as the evaluation of LASV qPCR assays.

To assess the field performance of ReLASV immunoassays in comparison to available qPCR assays we followed the guidance of Panning *et al*.^42^ who suggested that in the absence of BSL-4 capacity for live virus culture the results of RT-PCR based assays and enzyme immunoassay antigen detection may be combined to achieve a diagnostic standard. While PCR-based assays are often assumed to be more sensitive than immunoassays, the results of our current study indicate that this was not the case for samples from a recent cohort of Sierra Leonean patients with suspected Lassa fever. This highlights a critical need to formulate and implement a modern testing algorithm and a combined diagnostic standard to appropriately evaluate the sensitivity and specificity of LASV immunoassays and nucleic acid tests. Using a combined diagnostic standard, the sensitivity of qPCR assays was lower than that of either ReLASV immunoassay. The most robust qPCR assay (Nikisins) had a sensitivity of 82% compared to sensitivities of 95% for the ReLASV Ag-capture ELISA and 90% for the RDT. The immunoassays and qPCR assays both performed with high specificity, 95–100% under the combined diagnostic standard. It is unlikely that qPCR sensitivity is underestimated in the current study, and that the samples testing positive by the immunoassays but negative by qPCR are false positives. Samples from cases that were negative by qPCR, but positive by the ReLASV immunoassays, had a high CFR and an abnormal clinical chemistry profile typical of Lassa fever patients. The results of the current studies also suggest that the ReLASV RDT and Ag ELISA have increased sensitivity and specificity over previously developed LASV Ag-capture immunoassays^27^. Preliminary studies suggest that Pan Lassa RDTs based on polyclonal antibodies have increased sensitivity for detection of LASV of the divergent lineages compared to lineage IV ReLASV RDTs that are based on murine MAbs.

Since BSL-4 laboratories do not exist in the endemic Lassa fever zone, electrical power is sporadic and difficult to sustain, and as the ultimate goal of robust and field implementable diagnostics is the timely detection of viral protein antigen or RNA in suspected Lassa patients, thereby permitting immediate treatment, we propose when possible the ReLASV RDT should be used as a point-of-care test, followed by ELISA and qPCR as confirmatory assays. Data from this study and previous studies^1,28^ show that ReLASV tests are not prone to generating false positive results, and thus can serve as rule-in tests for Lassa fever and can inform the clinical management of Lassa fever patients. Detection of antigen by a ReLASV RDT should potentiate isolation and initial preparation of presumptive Lassa fever patients for treatment with ribavirin and supportive therapy, while ELISA and qPCR results are underway, which should require only an additional 2 to 3 hours, respectively, in settings such as the KGH and ISTH. Patients with acute Lassa fever who are antigenemic as determined by Ag ELISA or RDT may benefit from treatment with the antiviral drug ribavirin^1,22^. In the future, such patients may also benefit by treatment with Lassa fever drugs in the development pipeline, such as an immunotherapeutic cocktail that cured infected nonhuman primates more than a week after LASV challenge^18^ or small molecule drugs^45^. Immunoassays that measure anti-LASV IgM or IgG can also contribute to clinical management of Lassa fever patients. Previous studies indicate that survival of non-viremic IgM + cases is not improved by ribavirin treatment^1^. Patients at the post-acute stage of LASV infection whose immune response has controlled the LASV load, yet remain sick enough to seek treatment, may benefit from supportive therapies such as fluid replacement.

Widespread implementation of LASV RDTs could provide health care workers at remote PHUs with a reliable diagnostic tool rather than reliance on the less accurate non-specific clinical symptoms that have been the cornerstone of Lassa fever diagnosis for decades. Diagnosis using the ReLASV RDT is not dependent on sample processing, such as extraction of nucleic acids, or instrumentation that requires a stable source of electrical power as is the case with PCR. Reliance on central laboratory testing can delay VHF diagnosis by several days, resulting in increased time-to-treatment, and higher mortality rates. The performance characteristics of the ReLASV immunoassays indicate that they can reliably inform the diagnosis of LASV infected patients in the absence of nucleic acid based tests. Patients that test positive for Lassa fever by RDT should be transported to medical facilities where treatment with ribavirin and enhanced supportive care and infection control measures can occur.

The ReLASV RDT has utility in settings where nosocomial infections from Lassa fever patients pose a threat to health care workers. Since pregnant women are at increased risk for developing severe Lassa fever^1,14^, the use of LASV RDTs in maternity wards across the Lassa fever zone could aid in identification of potential cases and limit transmission. Likewise, surgical units or other settings in West African health care facilities where there is a high risk for exposure to blood or other bodily fluids would benefit by the use of LASV RDTs. Sporadic importation of Lassa fever cases to nations outside Africa in the nearly 50 years since identification of the febrile illness underscores the need for effective diagnostic and therapeutic preparedness in developed countries^46–48^. Human to human transmission of Lassa fever was recently reported in Germany^49^. The ease with which LASV can be procured and disseminated as a bioweapon further supports the need for development and stockpiling of diagnostics and other countermeasures by developed countries.

One caveat of the current studies is the relatively low numbers of Lassa fever subjects identified during the study period. This may reflect in part the reluctance of subjects to present to a VHF Ward in the aftermath of the 2013–16 Ebola outbreak. Not all Lassa patients could be included due to limitations of sample volume or lack of informed consent. Furthermore, a higher than expected CFR in the Lassa fever patients of 87% in the cohort may be due in part to the later presentation of cases to a health care provider than before the Ebola outbreak. Patients that present in the post-acute stage after initiation of anti-LASV humoral immune response would be expected to have lower levels of circulating virions, antigens and nucleic acids, which could impact the sensitivity of both PCR and antigen immunoassays. However, the emergence of anti-LASV IgM and IgG does not always correspond to waning of LASV nucleic acid or antigens. It is also pertinent to note that there is not uniform progression of the amount of LASV nucleic acid versus protein in the blood. In a subset of patients, LASV NP may appear earlier in the blood than LASV RNA. While the reverse situation was not observed in the current cohort, this situation has been observed in previous patients presenting to KGH. Further studies are needed to evaluate the performance of ReLASV RDT at the point-of-care and may establish the utility of the RDTs for earlier diagnosis. Development of more sensitive methods for detection of LASV genomic RNA should also proceed with an emphasis on platforms that are low cost, and can be deployed and maintained in austere environments.

The recent emergence of Ebola in West Africa illuminated the need for rapid diagnostics for high impact febrile illnesses^50^. As is the case with Lassa fever there exist serious deficiencies in the clinical diagnosis of Ebola. At one treatment center the WHO case definition for Ebola had a strikingly low specificity of 31.5%^51,52^. RDTs for Ebola were developed, but were not widely deployed during the West African Ebola outbreak^53^. Broadhurst and coworkers^54^ found that the recombinant Ebola RDT had 100% sensitivity and 92% specificity versus qPCR in a field setting. The specificity of the ReEBOV RDT was underestimated in this study because of false negative results of the qPCR assay used as the diagnostic standard^54^. The widespread use of Lassa and Ebola RDTs could identify Ebola should it re-emerge in West Africa thereby enabling a faster public health response, while addressing some of the challenges posed by the continuous threat of Lassa fever.

## Methods

### Study Design

Clinical research including all human subjects testing at KGH was approved by the Sierra Leone Ethics and Scientific Review Committee and the Tulane University Institutional Review Board (IRB). All research was performed in accordance with relevant guidelines/regulations. All subjects enrolled in this study and/or their legal guardians provided oral informed consent or written informed consent after the nature and possible consequences of the studies were explained. Only KGH staff were involved in the administration of health care to suspected Lassa fever patients at the KGH VHF Ward. All medical decisions, including whether or not to administer ribavirin to patients, were at the sole discretion of the attending KGH VHF Ward physician.

After patients were enrolled in this clinical study, a pre-admission evaluation was completed to determine if they met the case definition of a suspected Lassa case (Table S1)^12^. The suspected Lassa fever case definition was a reported or documented temperature ≥38 °C for less than three weeks with absence of local inflammation and at least 2 major signs, or 1 major plus 2 minor signs, or at least 3 minor signs. Contacts of suspected Lassa fever patients were also eligible for enrollment. Exclusion criteria included hemodynamic instability as determined by the treating physician.

Upon enrollment, patients received an admission history and physical exam on the Lassa Ward. Small blood volumes (typically 5 milliliters [mL]) for serum separation were collected in vacutainer tubes by experienced phlebotomists from study subjects after providing informed consent and were screened presumptively for Lassa fever in the VHF Laboratory using the ReLASV RDT, Ag-, IgM-, and IgG-ELISA and qPCR assays. Follow-up testing of subjects included blood draws on days two, three, four, seven, and ten. Testing on day ten corresponded to the final dose of ribavirin. Additional blood draws for follow-up testing were at the discretion of the treating physician. Long-term follow-up testing of suspected or convalescent cases included testing at days 30, 90, and 180.

### ReLASV Ag ELISA

The ReLASV Ag ELISA kit utilizes microwell plates coated with LASV NP-specific rabbit polyclonal antibody^28^. Patient serum is diluted 1:10 in sample buffer. Diluted reference, controls, and sample are transferred to the microwell plate (100 µL/well) and incubated for 60 minutes at 37 °C. Microwells are washed four times with 300 µL/well of a PBS-Tween wash solution using an automated ELISA plate washer. Peroxidase labeled LASV NP-specific rabbit polyclonal reagent is added to the microwells (100 µL/well) and incubated at ambient temperature for 30 minutes. Microwells are washed four times with 300 µL/well of PBS-Tween wash solution. TMB substrate (Moss, Inc. Pasadena, MD) is added (100 uL/well) and incubated for 10 minutes followed by stop solution (100 uL/well). Microplates are read at 450 nm with 650 nm subtraction with an OD450 nm cut-off of 0.097.

### ReLASV RDT

The ReLASV RDT (Lineage IV Josiah) has been developed using murine monoclonal antibodies specific for LASV nucleoprotein (NP) antigen^29,37^. The immunochromatographic dipstick design incorporates a plasma separator sample pad, a gold nanoparticle-labelled MAb, a test line consisting of a MAb that captures the LASV NP antigen-Mab nanoparticle complex, and a murine IgG specific control line. 30 µL of whole blood, plasma, or serum is introduced onto the sample pad and the dipstick is then inserted into a culture tube containing 200 µL (4 drops) of sample buffer, which initiates the flow of sample and sample buffer. Incubation time is 15–25 minutes at ambient temperature (18–30 °C) for full signal development. Results are scored on a scale of 0–5 using a visual aid. Signal intensity may be quantified by instrumentation. The KGH VHF laboratory performs reflectance measurements (mV) using the ESEQuant Lateral Flow Reader (QIAGEN Lake Constance GmbH, Germany) and calculates the ratio of Test Line (mV) to Control Line (mV) to normalize results for background staining.

### ReLASV IgM and IgG ELISA

The ReLASV IgM and IgG ELISA utilizes microwell plates coated with a mixture of ReLASV NP, glycoprotein complex (GPC), and Z matrix protein^28^. The reference, controls, and patient serum are diluted 1:100 in sample buffer. Diluted reference, controls, and samples are transferred into the microwell plate (100 µL/well) and incubated for 30 minutes at ambient temperature (18–30 °C). Microwells are washed four times with 300 µL/well of PBS-Tween wash solution. Peroxidase labeled human IgG or IgM Fc-specific caprine polyclonal reagent (Jackson ImmunoResearch Laboratories, Inc. West Grove, PA) is added to the microwells (100 µL/well) and incubated at ambient temperature for 30 minutes. Microwell wash step is repeated. A similar TMB substrate incubation is performed followed by addition of stop solution (100 uL/well). Microplates are read at 450 nm with 650 nm subtraction. The ReLASV IgM assay negative cut-off is OD = 0.226 with an Intermediate cut-off of OD = 0.452 (2X negative cut-off). The ReLASV IgG assay negative cut-off is OD = 0.170 with an intermediate cut-off of OD = 0.340 (2X negative cut-off). A calculated concentration approach was used to standardize the optical density values and negative control values as described in Dmitrienko *et al*. (2007). A four parameter logistic regression model was used to model the optical density values against their concentrations. Back calculations were applied to the four-parameter logistic model to express its estimated parameters as a function of the standardized optical density values. The crude optical density values were substituted into the back calculated equation to generate the standardized optical density values and negative control values. The cutoff for positivity was calculated according to 2.5 standard deviations above the mean standardized negative control value. For those cases where the algorithm for the four parameter logistic regression model did not converge, positivity was determined according to 2.5 standard deviations above the average negative control values for the entire data set.

### LASV quantitative polymerase chain reaction assays

Quantitative PCR using the LightCycler^®^96 System was used to evaluate the presence of viral genomes. Primer and probe sequences were obtained from previously published methods^33,41^. The Trombley qPCR assay targets the LASV glycoprotein gene with non-degenerate primers and probe. The Nikisins qPCR assay targets the LASV polymerase gene and utilizes a set of highly degenerate primers and probe. Clinical excess serum samples stored at −20 C were thawed at 4 C overnight. Viral RNA was extracted using QIAamp Viral RNA Mini Kit according to manufacturer’s instructions (Qiagen N.V., Germany) and stored at −20 C until analysis was performed, no more than 4 days post extraction. The amplification step of the qPCR assays was performed in a separate building from where RNA extractions from blood of suspected cases was performed. Both qPCR assays performed with high specificity, which may be attributed to scrupulous efforts to mitigate amplicon contamination in the KGH VHF laboratory.

TaqMan™ RNA-to-C_T_™ *1-Step* Kit (Applied Biosystems, Foster City, CA) was used according to manufacturer’s instructions with Trombley primers and probe specific to the Lassa Josiah nucleoprotein gene (F548 5′-GGA ATG AGT GGT GGT AAT CAA GG-3′, R617 5′-TTT TCA CAT CCC AAA CTC TCA CC-3′, p594A 6FAM-ACT CCA TCT CTC CCA GCC CGA GC-TAMRA-3′). Forward and reverse primers and the probe were used at 10 µM and added to the reaction mix at a volume of 1 µL, 1 µL, and 0.5 µL, respectively. 2 µL of viral RNA was added to the reaction mix resulting in a final volume of 20 uL. *Power* SYBR® Green RNA-to-CT™ 1-Step Kit (Applied Biosystems, Foster City, CA) was used according to manufacturer’s instructions with Nikisins primers (LaV F2 5′-CCA CCA TYT TRT GCA TRT gGCC A-3′, reverse LaV R 5′-GCA CAT GTN TCH TAY AGY ATG GAYC A-3′). Forward and reverse primers were used at 10 µM and added to the reaction mix at a volume of 1 µL each. 2 µL of viral RNA was added to the reaction mix resulting in a final volume of 20 uL. This SYBR based assay was directly compared to the version of the assay that uses a labeled probe. Both versions of the assay performed with high specificity, but the SYBR based assay was more sensitive, which appears to be due to sequence variation at the probe binding site in contemporary LASV strains. The RT-qPCR protocol consisted of a reverse transcription step (48 °C: 30 min and 95 °C:10 min) a two-step PCR amplification (94 °C:15 sec and 60 °C:30 sec for 45 cyccles) and finally a standard melting curve run on either the Lightcycler 96 System (Roche) or the 7900HT System (Applied Biosystems). To ensure the quality of the RNA extractions, we also performed 18 s RT-qPCR using HeLa genomic DNA as a standard.

### Clinical Chemistry

Samples were analyzed with a Piccolo Blood Chemistry Analyzer and Comprehensive Metabolic Reagent Discs (Abaxis), according to the manufacturer’s recommendations. We performed metabolic measurements that included the blood levels of sodium (Na), potassium (K), total carbon dioxide (tCO_2_), chloride (Cl), glucose (GLU), calcium (Ca), blood urea nitrogen (BUN), creatinine (CRE), alkaline phosphatase (ALP), alanine aminotransferase (ALT), aspartate aminotransferase (AST), total bilirubin (tBIL), albumin (ALB), and total protein (tPRO).

### Data Analysis and Statistical Methods

Laboratory data, including absorbance values were analyzed in their individual forms and were not transformed. Pairwise comparisons involving continuous variables were carried out on the ranks of the data values to account for any departures in normality or differences in standard deviations among comparison groups. Contingency analyses were performed with Fisher’s exact test. Logistic fit with Receiver Operator Curve (ROC) analysis was used to assess the diagnostic accuracy of the RDT. Data were analyzed using the SAS System (version 9.3, SAS Institute, Inc., Cary, NC). Analyses were two-tailed with a significance threshold set at p < 0.05.

## Electronic supplementary material

Supplementary Information
