# Supplementary material for: Field validation of recombinant antigen immunoassays for diagnosis of Lassa fever
Source: Sci Rep. 2018 Apr 12;8:5939. doi: 10.1038/s41598-018-24246-w (PMC5897328; doi:10.1038/s41598-018-24246-w)
Supplement: Supplementary file 1 — Supplementary Information [file 41598_2018_24246_MOESM1_ESM.docx]

**SUPPLEMENTARY INFORMATION**

**Field validation of recombinant antigen immunoassays for diagnosis of Lassa fever**

Matthew L. Boisen^1^, Jessica N. Hartnett^2^, Jeffrey G. Shaffer^3^, Augustine Goba^4,5^, Mambu Momoh^4,5,6^, John Demby Sandi^4,5^, Mohamed Fullah^4,5†^, Diana K. S. Nelson^1^, Duane J. Bush^1^ Megan M. Rowland^1^, Megan L. Heinrich^1^, Anatoliy P. Koval^1^, Robert W. Cross^7^, Kayla G. Barnes^8,9,10^, Anna E. Lachenauer^8,9^, Aaron E Lin^8,9^, Mahan Nekoui^8,9^, Dylan Kotliar^8,9^, Sarah M. Winnicki^8,9^, Katherine J. Siddle^8,9^, Michael Gbakie^4,5^, Mbalu Fonnie^4,5†^, Veronica J. Koroma^4,5^, Lansana Kanneh^4.5^ , Peter C. Kulakosky^11^, Kathryn M. Hastie^12^, Russell B. Wilson^11^, Kristian G. Andersen^12,13,14^, Onikepe O. Folarin^15^, Christian T. Happi^15,16^, Pardis C. Sabeti^8,9,10,17^, Thomas W. Geisbert^7^, Erica Ollmann Saphire^12,18^, S. Humarr Khan^4,5†^, Donald S. Grant^4,5^*, John S. Schieffelin^19^* **,** Luis M. Branco^1^***** and Robert F. Garry^1,2^*

^1^ Zalgen Labs, LCC, Germantown, MD USA 20876 and Aurora, CO, USA 80013

^2^ Tulane University, Department of Microbiology and Immunology, New Orleans, LA, 70112 USA

^3^ Tulane University, School of Public Health and Tropical Medicine, New Orleans, LA, 70112 USA

^4^ Viral Hemorrhagic Fever Program, Kenema Government Hospital, Kenema, Sierra Leone

^5^ Ministry of Health and Sanitation, Freetown, Sierra Leone

^6^ Eastern Polytechnic Institute, Kenema, Sierra Leone

^7^ University of Texas Medical Branch, Galveston National Laboratory, Galveston, TX, USA

^8^Center for Systems Biology, Department of Organismic and Evolutionary Biology, Harvard University, Cambridge, Massachusetts 02138, USA.

^9^The Broad Institute of MIT and Harvard, Cambridge, Massachusetts 02142, USA.

^10^Department of Immunology and Infectious Diseases, Harvard T.H. Chan School of Public Health, Harvard University, Boston, Massachusetts 02115, USA.

^11^ Autoimmune Technologies, LLC, New Orleans, LA, USA

^12^ Department of Immunology and Microbial Science, The Scripps Research Institute, La Jolla, California 92037, USA.

^13^Scripps Translational Science Institute, La Jolla, California 92037, USA.

^14^Department of Integrative Structural and Computational Biology, The Scripps Research Institute, La Jolla, California 92037, USA.

^15^Department of Biological Sciences, College of Natural Sciences, Redeemer's University, Ede, Osun State, Nigeria.

^16^African Center of Excellence for genomics of Infectious Diseases, Redeemer’s University, Ede, Osun State, Nigeria.

^17^Howard Hughes Medical Institute, Chevy Chase, Maryland 20815, USA.

^18^The Skaggs Institute for Chemical Biology, The Scripps Research Institute, LaJolla, CA, USA

^19^ Tulane University, Department of Pediatrics, Section of Infectious Diseases, New Orleans, LA, 70112 USA

**†**deceased

***Corresponding Authors**

Donald S. Grant, MBChB, MPH

Viral Hemorrhagic Fever Program,

Kenema Government Hospital

Kenema, Sierra Leone

donkumfel@yahoo.uk.co

John S. Schieffelin, MD. MSPH

Tulane University

Department of Pediatrics

Section of Infectious Diseases,

New Orleans, LA 70112

jschieff@tulane.edu

Luis M. Branco, PhD

Zalgen Labs

20271 Goldenrod Lane, Suite 2083

Germantown, MD 20876

[lbranco@zalgenlabs.com](mailto:lbranco@zalgenlabs.com)

Robert F. Garry, PhD

Department of Microbiology and Immunology

Tulane University School of Medicine

1430 Tulane Avenue, JBJ568

New Orleans, LA 70118

504-988-2027

[rfgarry@tulane.edu](mailto:rfgarry@tulane.edu)

Table S1. Suspected Lassa fever enrollment criteria: Major and minor Signs^1^

| **Major Signs** |
| --- |
| Bleeding (including IV injection sites) |
| Neck or facial edema |
| Conjunctivitis or sub-conjunctival hemorrhage |
| Spontaneous abortion or stillbirth |
| Petechial or hemorrhagic rash |
| Onset of tinnitus or altered hearing |
| Failure to respond within 48hrs to anti-malarial therapy  Failure to respond within 48hrs to antibiotic therapy |
| **Minor Signs** |
| Headache |
| Sore throat  Cough |
| Diarrhea  Vomiting |
| Chest/retrosternal pain |
| Weakness  Dizziness |
| Confusion  Convulsions |
| Diffuse abdominal pain/tenderness |
| Generalized myalgia or arthralgia |
| Jaundice |
| ^1^Modified from Khan et al. ^12^. The clinical signs case definition of suspected Lassa fever requires a reported or documented temperature ≥38°C for less than three weeks with absence of local inflammation and at least 2 major signs, or 1 major plus 2 minor signs, or at least 3 minor signs. |

| **Table S2. Performance of Lassa fever immunodiagnostics on blood samples from febrile patients meeting the case definition for suspected Lassa fever.** | | | | | | | |
| --- | --- | --- | --- | --- | --- | --- | --- |
| Sample  (hospital day) | ReLASV  Ag ELISA  (µg/ml)^1^ | ReLASV  RDT  (VS/RR)^2^ | ReLASV  IgM ELISA  (units/ml)^3^ | ReLASV  IgG ELISA  (units/ml)^4^ | Nikisins qPCR  (Ct)^5^ | Trombley qPCR  (Ct)^6^ | Outcome |
| G-6812-1 | positive (8.27) | positive  (3/0.28) | negative | negative | negative  (0) | negative  (0) | died |
| G-6835-1 | positive  (2.41) | positive  (4/ND^7^) | negative | negative | negative  (0) | negative  (0) | died |
| G-6835-2 | positive | ND | negative | negative | negative  (38.2)^8^ | negative  (0) |  |
| G-6869-1 | positive  (2.52) | positive  (2/0.33) | negative | negative | positive  (29.5) | ND | died |
| G-6899-1 | positive | positive  (3/0.50) | negative | negative | positive  (32.6) | ND | died |
| G-6899-2 | positive | positive  (5/1.02) | negative | negative | ND | ND |  |
| G-6956-1 | positive  (0.55) | positive  (3/0.47) | negative | negative | positive  (31.4) | ND | died |
| G-6956-2 | positive  (2.42) | positive  (4/0.55) | negative | negative | ND | ND |  |
| G-6956-3 | positive  (0.80) | positive  (ND) | negative | negative | negative  (37.5)^8^ | positive  (32.4) |  |
| G-6984-1 | positive  (0.94) | positive  (4/0.46) | ND | ND | ND | ND | died |
| G-6984-2 | positive  (1.36) | positive  (4/0.69) | ND | ND | ND | ND |  |
| G-6984-3 | ND | negative | ND | ND | ND | ND |  |
| G-6984-4 | negative | negative | ND | ND | ND | ND |  |
| G-6984-11 | negative | negative | ND | ND | positive  (37.0) | positive  (36.8) |  |
| G-7022-1 | positive | positive  (4/0.79) | negative | negative | positive  (32.7) | positive  (35.0) | died |
| G-7022-2 | positive  (1.10) | positive  (4/0.68) | negative | negative | ND | ND |  |
| G-7022-3 | positive | positive  (4/0.48) | negative | negative | positive  (34.5) | negative  (36.2)^8^ |  |
| G-7022-4 | positive | positive  (2/0.15) | ND | negative | negative  (36.0) | negative  (38.9)^8^ |  |
| G-7030-1 | positive | positive  (3/0.36) | negative | negative | ND | ND | died |
| G-7030-2 | positive  (0.71) | positive  (ND) | negative | negative | positive  (34.7) | positive  (30.0) |  |
| G-7030-3 | positive | positive  (ND) | positive | negative | positive  (36.0) | positive  (31.5) |  |
| G-7030-4 | positive | negative | positive | positive | positive  (34.0) | positive  (28.9) |  |
| G-7030-7 | negative | negative | positive  (27.8) | positive | negative  (0) | negative  (0) |  |
| G-7030-10 | negative | ND | positive  (22.9) | positive  (76.1) | negative  (0) | negative  (0) |  |
| G-7039-1 | negative | negative | positive | positive | ND | ND | discharged |
| G-7039-2 | negative | negative | positive | negative | negative  (0) | negative  (0) |  |
| G-7039-3 | negative | negative | positive | positive | negative  (0) | negative  (0) |  |
| G-7039-6 | negative | negative | positive | positive | negative  (0) | negative  (0) |  |
| G-7043-1 | negative | negative | positive | negative | negative  (39.2)^8^ | ND | Not admitted |
| G-7086-1 | positive  (1.80) | positive  (5/0.87) | positive | negative | ND | ND | died |
| G-7086-2 | positive  (1.68) | positive  (1/0.24) | positive | negative | positive  (36.4) | positive  (28.3) |  |
| G-7086-3 | positive | negative | positive | negative | positive  (32.9) | positive  (25.5) |  |
| G-7100-1 | negative | negative | negative | negative | positive  (32.3) | ND | unknown |
| G-7107-1 | positive  (2.89) | positive  (5/1.03) | negative | negative | positive  (34.4) | negative  (0) | died |
| G-7108-1 | positive  (1.27) | positive  (5/0.74) | positive | negative | positive  (30.0) | positive  (26.0) | died |
| G-7112-1 | positive  (0.27) | positive  (5/1.02) | negative | negative | positive  (26.2) | ND | died |
| G-7140-1 | positive  (4.26) | positive  (1/0.04) | positive  (39.4) | positive  (24.5) | ND | ND | died |
| G-7140-2 | positive | positive  (1/0.04) | negative | positive | positive  (30.2) | positive  (29.2) |  |
| G-7140-3 | positive | negative | negative | positive  (104.7) | positive  (29.2) | positive  (32.4) |  |
| G-7140-4 | positive  (0.25) | negative | negative | positive  (79.5) | ND | ND |  |
| G-7167-1 | positive  (0.54) | positive  (4/0.45) | negative | positive  (7.98) | ND | ND | died |
| G-7167-2 | positive  (0.33) | positive  (2/0.14) | negative | positive  (12.81) | positive  (37.0) | positive  (33.3) |  |
| G-7167-3 | positive  (0.27) | ND | ND | ND | positive  (32.8) | positive  (29.3) |  |
| G-7167-4 | negative | negative | negative | positive  (39.39) | ND | ND |  |
| G-7167-9 | negative | negative | negative. | positive  (72.87) | ND | ND |  |
| G-7167-13 | negative | negative | negative | positive  (151.14) | ND | ND |  |
| G-7197-1 | positive  (0.84) | positive  (4/0.52) | negative | negative | ND | ND | died |
| G-7197-2 | positive | positive  (4/0.43) | negative | negative | positive  (31.0) | positive  (32.4) |  |
| G-7197-3 | positive | positive  (4/0.40) | negative | negative | negative  (0) | negative  (0) |  |
| G-7197-9 | negative | ND | negative | negative | ND | ND |  |
| G-7197-16 | negative | negative | positive | positive  (28.98) | negative  (0) | negative  (0) |  |
| G-7218-1 | positive | positive  (4/0.45) | negative | negative | ND | ND | died |
| G-7218-2 | positive | positive  (4/0.45) | negative | negative | positive  (33.2) | ND |  |
| G-7254-1 | positive (0.30) | positive  (1/0.06) | ND | ND | negative  (39.6)^8^ | positive (29.6) | died |
| G-7254-2 | ND | positive  (4/0.97) | ND | ND | negative  (39.5)^8^ | negative  (37.2)^8^ |  |
| G-7254-3 | positive  (2.27) | positive  (3/0.86) | ND | ND | ND | ND |  |
| G-7254-4 | ND | positive  (5/0.84) | ND | ND | positive (36.6) | negative  (37.1)^8^ |  |
| G-7260-1 | positive  (0.69) | positive  (3/0.63) | ND | ND | positive  (31.8) | ND | died |
| G-7277-1a | negative | negative | positive | positive  (20.1) | ND | ND | discharged |
| G-7277-1b | negative | negative | positive | positive | negative  (43.3)^8^ | negative  (0) |  |
| G-7277-2 | negative | negative | positive | positive | negative  (0) | negative  (0) |  |
| G-7277-4 | negative | negative | positive | positive  (23.18) | ND | ND |  |
| G-7277-7 | negative | negative | positive | positive  (20.46) | ND | ND |  |
| G-7277-9 | negative | negative | positive | positive  (18.87) | ND | ND |  |
| G-7277-31 | negative  (0.00) | negative | positive  (33.23) | positive  (31.27) | ND | ND |  |
| G-7284-1 | positive | positive  (4/0.75) | negative | negative | negative  (39.9)^8^ | negative  (0) | died |
| G-7284-2 | positive  (0.07) | positive  (3/0.25) | negative | negative | negative  (0) | negative  (37.9)^8^ |  |
| G-7284-3 | positive  (0.03) | negative | negative | negative | positive (36.6) | positive (33.7) |  |
| G-7291-1 | positive | positive  (5/1.02) | negative | negative | positive (28.2) | ND | died |
| G-7309-1 | positive  (1.78) | positive  (1.06) | negative | negative | positive (26.5) | negative  (0) | died |
| G-7309-2 | positive | positive  (1.02) | negative | negative | ND | ND |  |
| G-7356-1 | positive  (0.77) | positive  (3/0.41) | negative | negative | negative  (0) | negative  (0) | died |
| G-7356-2 | positive  (0.40) | ND | negative | negative | ND | ND |  |
| G-7360-1 | positive | positive  (3/ND) | ND | ND | positive (34.0) | ND | died |
| G-7400-1 | positive | positive  (4/0.77) | ND | ND | positive (30.2) | ND | died |
| G-7428-1 | negative | negative | ND | ND | negative  (0) | negative  (0) | Not admitted |
| G-7494-1 | positive | positive  (3/0.37) | negative | negative | ND | ND | died |
| G-7494-2 | positive  (1.42) | positive  (3/0.34) | negative | negative | positive  (23.4) | negative  (0) |  |
| G-7494-3 | negative | negative | negative | negative | ND | ND |  |
| G-7494-4 | negative | negative | negative | negative | ND | ND |  |
| G-7501-1 | negative | negative | negative | negative | negative  (0) | negative  (0) | Not admitted |
| G-7502-1 | positive | positive  (4/0.85) | negative | negative | positive  (25.4) | positive  (30.2) | died |
| G-7502-2 | positive | positive  (4/0.45) | negative | negative | positive  (23.2) | positive  (28.3) |  |
| G-7504-1 | negative | negative | negative | negative | negative  (0) | negative  (0) | Not admitted |
| G-7506-1 | negative | negative | negative | negative | negative  (0) | negative  (0) | Not admitted |
| G-7508-1 | positive | positive  (1/ND) | positive | negative | positive (26.8) | positive (27.5) | Unknown |
| G-7516-1 | negative | negative | negative | positive  (15.3) | negative  (0) | negative  (0) | discharged |
| G-7516-2 | negative | ND | negative | positive  (13.4) | negative  (0) | negative  (0) |  |
| G-7522-1 | negative | negative | negative  (3.99) | positive  (22.7) | negative  (0) | negative  (0) | Not admitted |
| G-7524-1 | positive | positive  (1/0.10) | negative | negative | negative  (0) | negative  (0) | died |
| G-7524-2 | positive | ND | negative | positive | positive (33.8) | negative  (0) |  |
| G-7526-1 | positive  (2.94) | positive  (5/0.86) | negative  (0.42) | negative | negative  (41.8)^8^ | negative  (0) | died |
| G-7530-1 | positive | positive  (4/0.33) |  | negative | positive  (32.5) | positive  (29.0) | died |
| G-7530-2 | positive | positive  (3/0.33) | negative | negative | positive  (32.5) | positive  (29.0) |  |
| G-7530-3 | positive  (0.37) | positive  (3/0.23) |  | negative | ND | ND |  |
| G-7583-1 | positive  (0.09) | positive  (3/0.39) | negative | negative | positive  (27.0) | positive  (20.0) | discharged |
| G-7583-2 | positive | positive  (2/0.13) | negative | negative | positive  (24.0) | positive  (20.0) |  |
| G-7583-3 | ND | positive  (1/0.12) | ND | ND | ND | ND |  |
| G-7583-4 | negative | negative | negative | negative | ND | ND |  |
| G-7583-7 | negative | negative | positive | negative | ND | ND |  |
| G-7583-10 | negative | negative | positive | positive | ND | ND |  |
| G-7583-40 | negative | negative | positive | positive | ND | ND |  |
| G-7584-1 | positive | negative | negative | negative | positive  (24.5) | positive  (27.5) | died |
| G-7584-2a | positive  (2.42) | positive  (1/ND) | negative | negative | positive  (22.3) | positive  (27.0) |  |
| G-7584-2b | positive  (0.31) | ND | negative | negative | ND | ND |  |
| G-7594-1 | negative | negative | negative | negative | negative  (0) | negative  (0) | Not admitted |
| G-7599-1 | negative | negative | negative | negative | negative  (0) | negative  (0) | Not admitted |
| G-7600-1 | negative | negative | negative | negative | negative  (0) | negative  (0) | Not admitted |
| G-7601-1 | positive  (0.81) | positive  (5/0.62) | negative | negative | negative (40.7)^8^ | negative  (0) | died |
| G-7601-2 | positive  (2.36) | ND | negative | negative | negative (37.7)^8^ | negative  (0) |  |
| G-7601-3 | positive  (2.05) | positive  (5/0.49) | negative | negative | negative (38.9)^8^ | negative  (0) |  |
| G-7601-4 | negative | positive  (2/0.28) | negative | negative | ND | ND |  |
| G-7603-1 | negative | negative | negative | negative | negative  (0) | negative  (0) | not admitted |
| G-7605-1 | negative | negative | negative | negative | negative  (0) | negative  (0) | not admitted |
| G-7615-1 | positive  (0.25) | negative^9^ | positive | negative | negative (41.8)^8^ | negative  (0) | died |
| G-7617-1 | positive  (1.05) | positive  (2/0.14) | negative | negative | negative (43.7)^8^ | negative  (0) | discharged |
| G-7617-3 | positive  (0.43) | ND | negative | negative | negative (43.2)^8^ | negative  (0) |  |
| G-7617-4 | positive  (0.42) | negative | negative | negative | negative  (0) | negative  (0) |  |
| G-7617-5 | positive  (0.13) | negative | negative | negative | negative (43.6)^8^ | negative  (0) |  |
| G-7617-8 | negative | negative | negative | negative | negative  (0) | ND |  |
| G-7617-11 | negative | negative | negative | negative | negative (43.2)^8^ | negative  (0) |  |
| G-7619-1 | positive  (0.13) | positive  (1/0.09) | negative | negative | negative  (0) | negative  (0) | discharged |
| G-7619-2 | positive  (0.43) | ND | negative | negative | negative  (0) | negative  (0) |  |
| G-7619-3 | positive  (0.42) | negative | negative | negative | positive  (36.6) | negative  (0) |  |
| G-7619-4 | positive  (0.30) | negative | negative | negative | negative  (0) | negative  (0) |  |
| G-7619-7 | negative | negative | negative | negative | negative  (39.9)^8^ | negative  (0) |  |
| G-7619-10 | negative | negative | negative | negative | negative  (0) | negative  (0) |  |
| G-7620-1 | negative | negative | negative | negative | negative  (0) | ND | not admitted |
| G-7621-1 | negative | negative | negative | negative | negative  (0) | ND | not admitted |
| G-7627-1 | negative | negative | negative | positive | negative  (0) | ND | not admitted |
| G-7629-1 | negative | negative | negative | negative | negative  (0) | ND | not admitted |
| G-7630-1 | negative | negative | negative | negative | negative  (0) | ND | not admitted |
| G-7632-1 | negative | negative | negative | negative | negative  (41.5)^8^ | ND | not admitted |
| G-7633-1 | negative | negative | negative | negative | negative  (40.4)^8^ | ND | not admitted |
| G-7634-1 | negative | negative | negative | negative | negative  (0) | ND | not admitted |
| G-7635-1 | negative | negative | negative | negative | negative  (39.7)^8^ | ND | not admitted |
| G-7636-1 | negative | negative | negative | negative | negative  (41.1)^8^ | ND | not admitted |
| G-7637-1 | negative | negative | negative | negative | negative  (37.5)^8^ | ND | not admitted |
| G-7638-1 | positive  (0.72) | positive  (2/0.18) | negative | negative | positive  (36.5) | ND | discharged |
| G-7638-2 | positive  (1.90) | positive  (3/0.38) | negative | negative | ND | ND |  |
| G-7638-3 | positive  (0.60) | positive  (1/0.05) | negative | negative | ND | ND |  |
| G-7638-4 | positive  (0.39) | negative | negative | negative | ND | ND |  |
| G-7638-7 | positive  (0.09) | negative | negative | negative | ND | ND |  |
| G-7638-10 | negative | negative | negative | negative | ND | ND |  |
| G-7642-1 | negative | negative | negative | negative | negative  (41.8)^2^ | ND | not admitted |
| G-7645-1 | negative | negative | negative | negative | negative  (0) | ND | not admitted |
| G-7649-1 | negative | negative | negative | negative | negative  (0) | ND | not admitted |
| G-7652-1 | negative | negative | negative | negative | negative  (0) | ND | not admitted |
| G-7661-1 | positive  (0.49) | negative^9^ | positive  (28.01) | positive  (31.17) | positive  (30.2) | ND | died |
| G-7661-2 | positive  (0.67) | negative | positive  (42.63) | positive  (26.24) | positive (28.4) | ND |  |
| G-7663-1 | negative | negative | positive  (26.91) | positive  (68.15) | negative  (43.1)^8^ | ND | not admitted |
| G-7664-1 | negative | negative | negative | positive  (58.61) | negative  (0) | ND | not admitted |
| G-7665-1 | negative | negative | negative | negative | negative  (0) | ND | not admitted |
| G-7666-1 | negative | negative | negative | positive  (30.9) | negative  (0) | ND | not admitted |
| G-7669-1 | positive  (4.72) | positive  (5/0.92) | positive  (12.80) | negative | positive (23.1) | ND | died |
| G-7669-2 | positive  (2.61) | ND | positive  (10.90) | negative | ND | ND |  |
| G-7672-1 | negative | negative | negative | negative | negative  (0) | ND | not admitted |
| G-7674-1 | negative | negative | positive  (24.75) | positive  (25.67) | positive (30.0) | ND | died |
| G-7675-1 | negative | negative | positive  (21.10) | negative | negative  (38.5)^8^ | ND | not admitted |
| G-7684-1 | negative | negative | negative | negative | negative  (0) | ND | discharged |
| G-7684-3 | negative | negative | negative | negative | negative  (0) | ND |  |
| G-7684-5 | negative | negative | negative | negative | ND | ND |  |
| G-7684-6 | negative | negative | negative | negative | ND | ND |  |
| ^1^antigen levels (µg/ml) values below cutoff (0.3 µg/ml) are not indicated  ^2^VS=visual score (0-5), RR=reflectance ratio. VS zero scores and RR values below cutoff (0.02) are not indicated  ^3^IgM levels (relative unit/ml). Values below cutoff (13.8 units/ml) are not indicated.  ^4^IgG levels (relative unit/ml). Values below cutoff (6.0 units/ml) are not indicated  ^5^Ct=cycle threshold. Cutoff=37  ^6^Ct=cycle threshold. Cutoff=35  ^7^ ND=not done  ^8^above cutoff  ^9^sample was positive on prototype Pan Lassa RDT | | | | | | | |

**Table S3. Receiver operator Curve calculation of Nikisins and Trombley quantitative polymerase chain reaction assay cutoff.**

Tombley qPCR

| **X** | **Prob** | | **1-Specificity** | | **Sensitivity** | | **Sens-(1-Spec)** | |  | **True Pos** | | | **True Neg** | | **False Pos** | | **False Neg** | |  |
| --- | --- | --- | --- | --- | --- | --- | --- | --- | --- | --- | --- | --- | --- | --- | --- | --- | --- | --- | --- |
| . | . | | 0.0000 | | 0.0000 | | 0.0000 | |  | 0 | | | 39 | | 0 | | 38 | |  |
| 23.10000 | 0.9933 | | 0.0000 | | 0.0263 | | 0.0263 | |  | 1 | | | 39 | | 0 | | 37 | |  |
| 23.40000 | 0.9926 | | 0.0000 | | 0.0526 | | 0.0526 | |  | 2 | | | 39 | | 0 | | 36 | |  |
| 24.50000 | 0.9896 | | 0.0000 | | 0.0789 | | 0.0789 | |  | 3 | | | 39 | | 0 | | 35 | |  |
| 25.40000 | 0.9862 | | 0.0000 | | 0.1053 | | 0.1053 | |  | 4 | | | 39 | | 0 | | 34 | |  |
| 26.20000 | 0.9823 | | 0.0000 | | 0.1316 | | 0.1316 | |  | 5 | | | 39 | | 0 | | 33 | |  |
| 26.50000 | 0.9806 | | 0.0000 | | 0.1579 | | 0.1579 | |  | 6 | | | 39 | | 0 | | 32 | |  |
| 26.80000 | 0.9787 | | 0.0000 | | 0.1842 | | 0.1842 | |  | 7 | | | 39 | | 0 | | 31 | |  |
| 27.00000 | 0.9773 | | 0.0000 | | 0.2105 | | 0.2105 | |  | 8 | | | 39 | | 0 | | 30 | |  |
| 28.20000 | 0.9672 | | 0.0000 | | 0.2368 | | 0.2368 | |  | 9 | | | 39 | | 0 | | 29 | |  |
| 29.50000 | 0.9514 | | 0.0000 | | 0.2632 | | 0.2632 | |  | 10 | | | 39 | | 0 | | 28 | |  |
| 30.00000 | 0.9436 | | 0.0256 | | 0.2895 | | 0.2638 | |  | 11 | | | 38 | | 1 | | 27 | |  |
| 30.20000 | 0.9401 | | 0.0256 | | 0.3684 | | 0.3428 | |  | 14 | | | 38 | | 1 | | 24 | |  |
| 31.00000 | 0.9243 | | 0.0256 | | 0.3947 | | 0.3691 | |  | 15 | | | 38 | | 1 | | 23 | |  |
| 31.40000 | 0.9149 | | 0.0256 | | 0.4211 | | 0.3954 | |  | 16 | | | 38 | | 1 | | 22 | |  |
| 31.80000 | 0.9046 | | 0.0256 | | 0.4474 | | 0.4217 | |  | 17 | | | 38 | | 1 | | 21 | |  |
| 32.30000 | 0.8901 | | 0.0513 | | 0.4474 | | 0.3961 | |  | 17 | | | 37 | | 2 | | 21 | |  |
| 32.50000 | 0.8837 | | 0.0513 | | 0.4737 | | 0.4224 | |  | 18 | | | 37 | | 2 | | 20 | |  |
| 32.60000 | 0.8805 | | 0.0513 | | 0.5000 | | 0.4487 | |  | 19 | | | 37 | | 2 | | 19 | |  |
| 32.70000 | 0.8771 | | 0.0513 | | 0.5263 | | 0.4750 | |  | 20 | | | 37 | | 2 | | 18 | |  |
| 33.20000 | 0.8591 | | 0.0513 | | 0.5526 | | 0.5013 | |  | 21 | | | 37 | | 2 | | 17 | |  |
| 33.80000 | 0.8345 | | 0.0513 | | 0.5789 | | 0.5277 | |  | 22 | | | 37 | | 2 | | 16 | |  |
| 34.00000 | 0.8256 | | 0.0513 | | 0.6053 | | 0.5540 | |  | 23 | | | 37 | | 2 | | 15 | |  |
| 34.40000 | 0.8067 | | 0.0513 | | 0.6316 | | 0.5803 | |  | 24 | | | 37 | | 2 | | 14 | |  |
| 34.70000 | 0.7915 | | 0.0513 | | 0.6579 | | 0.6066 | |  | 25 | | | 37 | | 2 | | 13 | |  |
| 36.40000 | 0.6895 | | 0.0513 | | 0.6842 | | 0.6329 | |  | 26 | | | 37 | | 2 | | 12 | |  |
| 36.50000 | 0.6827 | | 0.0513 | | 0.7105 | | 0.6592 | |  | 27 | | | 37 | | 2 | | 11 | |  |
| 36.60000 | 0.6758 | | 0.0513 | | 0.7632 | | 0.7119 | |  | 29 | | | 37 | | 2 | | 9 | |  |
| 37.00000 | 0.6476 | | 0.0769 | | 0.7895 | | 0.7126 | | * | 30 | | | 36 | | 3 | | 8 | |  |
| 37.50000 | 0.6108 | | 0.1026 | | 0.7895 | | 0.6869 | |  | 30 | | | 35 | | 4 | | 8 | |  |
| 38.50000 | 0.5337 | | 0.1282 | | 0.7895 | | 0.6613 | |  | 30 | | | 34 | | 5 | | 8 | |  |
| 39.20000 | 0.4786 | | 0.1538 | | 0.7895 | | 0.6356 | |  | 30 | | | 33 | | 6 | | 8 | |  |
| 39.60000 | 0.4472 | | 0.1538 | | 0.8158 | | 0.6619 | |  | 31 | | | 33 | | 6 | | 7 | |  |
| 39.70000 | 0.4394 | | 0.1795 | | 0.8158 | | 0.6363 | |  | 31 | | | 32 | | 7 | | 7 | |  |
| 40.40000 | 0.3860 | | 0.2051 | | 0.8158 | | 0.6107 | |  | 31 | | | 31 | | 8 | | 7 | |  |
| 40.70000 | 0.3638 | | 0.2051 | | 0.8421 | | 0.6370 | |  | 32 | | | 31 | | 8 | | 6 | |  |
| 41.10000 | 0.3351 | | 0.2308 | | 0.8421 | | 0.6113 | |  | 32 | | | 30 | | 9 | | 6 | |  |
| 41.50000 | 0.3076 | | 0.2564 | | 0.8421 | | 0.5857 | |  | 32 | | | 29 | | 10 | | 6 | |  |
| 41.80000 | 0.2878 | | 0.2821 | | 0.8947 | | 0.6127 | |  | 34 | | | 28 | | 11 | | 4 | |  |
| 43.10000 | 0.2114 | | 0.3077 | | 0.8947 | | 0.5870 | |  | 34 | | | 27 | | 12 | | 4 | |  |
| 43.30000 | 0.2011 | | 0.3333 | | 0.8947 | | 0.5614 | |  | 34 | | | 26 | | 13 | | 4 | |  |
| 43.70000 | 0.1816 | | 0.3333 | | 0.9211 | | 0.5877 | |  | 35 | | | 26 | | 13 | | 3 | |  |
| 45.00000 | 0.1283 | | 1.0000 | | 1.0000 | | 0.0000 | |  | 38 | | | 0 | | 39 | | 0 | |  |
| 45.00000 | 0.1283 | | 1.0000 | | 1.0000 | | 0.0000 | |  | 38 | | | 0 | | 39 | | 0 | |  |
| Trombley qPCR |  | |  | |  | |  | |  |  | | |  | |  | |  | |  |
| **X** | **Prob** | | **1-Specificity** | | **Sensitivity** | | **Sens-(1-Spec)** | |  | **True Pos** | | | **True Neg** | | **False Pos** | | **False Neg** | |  |
| . | . | | 0.0000 | | 0.0000 | | 0.0000 | |  | 0 | | | 14 | | 0 | | 26 | |  |
| 20.00000 | 0.9967 | | 0.0000 | | 0.0385 | | 0.0385 | |  | 1 | | | 14 | | 0 | | 25 | |  |
| 26.00000 | 0.9868 | | 0.0000 | | 0.0769 | | 0.0769 | |  | 2 | | | 14 | | 0 | | 24 | |  |
| 27.50000 | 0.9814 | | 0.0000 | | 0.1538 | | 0.1538 | |  | 4 | | | 14 | | 0 | | 22 | |  |
| 28.30000 | 0.9777 | | 0.0000 | | 0.1923 | | 0.1923 | |  | 5 | | | 14 | | 0 | | 21 | |  |
| 29.00000 | 0.9738 | | 0.0000 | | 0.2308 | | 0.2308 | |  | 6 | | | 14 | | 0 | | 20 | |  |
| 29.20000 | 0.9726 | | 0.0000 | | 0.2692 | | 0.2692 | |  | 7 | | | 14 | | 0 | | 19 | |  |
| 29.60000 | 0.9699 | | 0.0000 | | 0.3077 | | 0.3077 | |  | 8 | | | 14 | | 0 | | 18 | |  |
| 30.00000 | 0.9671 | | 0.0000 | | 0.3462 | | 0.3462 | |  | 9 | | | 14 | | 0 | | 17 | |  |
| 30.20000 | 0.9656 | | 0.0000 | | 0.3846 | | 0.3846 | |  | 10 | | | 14 | | 0 | | 16 | |  |
| 32.40000 | 0.9437 | | 0.0000 | | 0.4231 | | 0.4231 | |  | 11 | | | 14 | | 0 | | 15 | |  |
| 33.30000 | 0.9313 | | 0.0000 | | 0.4615 | | 0.4615 | |  | 12 | | | 14 | | 0 | | 14 | |  |
| 33.70000 | 0.9251 | | 0.0000 | | 0.5000 | | 0.5000 | |  | 13 | | | 14 | | 0 | | 13 | |  |
| 35.00000 | 0.9011 | | 0.0000 | | 0.5385 | | 0.5385 | | * | 14 | | | 14 | | 0 | | 12 | |  |
| 36.80000 | 0.8566 | | 0.0714 | | 0.5385 | | 0.4670 | |  | 14 | | | 13 | | 1 | | 12 | |  |
| 45.00000 | 0.4668 | | 1.0000 | | 1.0000 | | 0.0000 | |  | 26 | | | 0 | | 14 | | 0 | |  |
| 45.00000 | 0.4668 | | 1.0000 | | 1.0000 | | 0.0000 | |  | 26 | | | 0 | | 14 | | 0 | |  |
| * calculated cutoffs | |  | |  | |  | |  | | |  |  | |  | |  | |  | |

| **Table S4.** | | | | | | | | | | | | | | | |
| --- | --- | --- | --- | --- | --- | --- | --- | --- | --- | --- | --- | --- | --- | --- | --- |
| group | n | Na  mmol/L | K  mmol/L | tCO2  mmol/L | Cl  mmol/L | Glu  mg/dL | Ca  mg/dL | BUN  mg/dL | CRE  mg/dL | ALP  U/L | ALT  U/L | AST  U/L | tBIL  mg/dL | ALB  g/dL | tPRO  g/dL |
| True  Pos* | 14 | 131  ±2.4 | 5.5  ±0.4 | 14.9  ±1.3 | 97.2  ±2.4 | 89.6  ±13.6 | 6.8  ±0.4 | 49.3  ±8.7  p=.0005 | 4.3  ±0.9  p=.0001 | 472  ±103  p=.0001 | 1042  ±162  p<0001 | 1871  ±106  p<0001 | 1.28  ±0.2  p=0009 | 2.7  ±0.4  p=031 | 6.1  ±0.3  p=007 |
| False  Neg qPCR | 8 | 143  ±7.8 | 6.4  ±0.6 | 11.1  ±1.4  p=.026 | 92.4  ±2.7 | 82.4  ±15.1 | 6.0  ±0.6 | 21.8  ±3.6 | 2.1  ±0.5  p=.036 | 304  ±101  p=.008 | 685  ±144  p<0001 | 2000  ±0  p<0001 | 1.9  ±0.7  p=005 | 2.3  ±0.2  p=017 | 6.5  ±0.3 |
| True Neg | 21 | 139  ±3.2 | 6.2  ±0.4 | 16.9  ±1.6 | 99.6  ±2.5 | 128  ±37.0 | 6.3  ±0.5 | 22.7  ±9.0 | 0.9  ±0.1 | 57.6  ±14.7 | 52.6  ±13.7 | 118  ±48 | 0.9  ±0.3 | 3.1  ±0.2 | 7.3  ±0.3 |

* Values are averages ± SEM. Significant difference if observed versus True Negative samples is indicated.

|  |  |  |  |  |  |
| --- | --- | --- | --- | --- | --- |
| **Table S5. Repeat of negative quantitative polymerase chain reaction samples at 1:10 dilution** | | | | | |
| **Sample number** | **ReLASV Ag ELISA** | **ReLASV RDT** | **Initial Nikisins qPCR Ct**  **1:1** | **Repeat Nikisins qPCR Ct**  **1:1** | **Repeat Nikisins qPCR Ct**  **1:10** |
|  |  |  |  |  |  |
| G-7601-1 | positive | positive | 40.7^1^ | 0 | 0 |
| G-7615-1 | positive | negative^2^ | 41.8^1^ | 0 | 0 |
| G-7617-1 | positive | positive | 43.7^1^ | 39.4^1^ | 40.7^1^ |
|  |  |  |  |  |  |
|  |  |  |  |  |  |

^1^above cutoff

^2^G-7615-1 was positive on Pan Lassa RDT.

| **Table S6. Performance of Lassa fever immunodiagnostics on blood samples from consented contacts of Lassa fever patients.** | | | | | | |
| --- | --- | --- | --- | --- | --- | --- |
| Sample | ReLASV  Ag ELISA | ReLASV  RDT | ReLASV  IgM ELISA | ReLASV  IgG ELISA | Nikisins qPCR  (Ct) | Trombley qPCR  (Ct) |
| G-6856 | positive | positive | negative | negative | positive  (33.0) | positive  (27.4) |
| G-7147 | negative | negative | negative | negative | negative  (0) | negative  (0) |
| G-7148 | negative | negative | negative | negative | negative  (0) | negative  (0) |
| G-7149 | negative | negative | negative | negative | negative  (0) | negative  (0) |
| G-7198 | negative | negative | negative | negative | negative  (0) | negative  (0) |
| G-7206 | negative | negative | negative | negative | negative  (0) | negative  (0) |
| G-7207 | negative | negative | negative | negative | negative  (0) | negative  (0) |
| G-7208 | negative | negative | negative | negative | negative  (0) | negative  (0) |
| G-7247 | negative | negative | negative | negative | negative  (0) | negative  (0) |
| G-7248 | negative | negative | negative | negative | negative  (0) | negative  (0) |
| G-7286 | negative | negative | negative | negative | negative  (0) | negative  (0) |
| G-7287 | negative | negative | negative | negative | negative  (0) | negative  (0) |
| G-7288 | negative | negative | negative | negative | negative  (0) | negative  (0) |
| G-7289 | negative | negative | negative | negative | negative  (0) | negative  (0) |
| G-7290 | negative | negative | negative | negative | negative  (0) | negative  (0) |
| G-7305 | negative | negative | negative | negative | negative  (0) | negative  (0) |
| G-7319 | negative | negative | negative | negative | negative  (0) | negative  (0) |
| G-7321 | negative | negative | negative | negative | negative  (0) | negative  (0) |
| G-7322 | negative | negative | negative | negative | negative  (0) | negative  (0) |
| G-7323 | negative | negative | negative | negative | negative  (0) | negative  (0) |
| G-7324 | negative | negative | negative | negative | negative  (0) | negative  (0) |
| G-7325 | negative | negative | negative | negative | negative  (0) | negative  (0) |
| G-7326 | negative | negative | negative | negative | negative  (0) | negative  (0) |
| G-7327 | negative | negative | negative | negative | negative  (0) | negative  (0) |
| G-7328 | negative | negative | negative | negative | negative  (0) | negative  (0) |

| **Table S7. Performance of Lassa fever immunodiagnostics on blood samples from nonAfrican controls.** | | | | | |
| --- | --- | --- | --- | --- | --- |
| Sample | ReLASV  Ag ELISA | ReLASV  RDT | ReLASV  IgM ELISA | ReLASV  IgG ELISA | Nikisins qPCR  (Ct) |
| B19 1 | negative | negative | negative | negative | negative  (0) |
| B19 2 | negative | negative | negative | negative | negative  (0) |
| B19 3 | negative | negative | negative | negative | negative  (0) |
| B19 4 | negative | negative | negative | negative | negative  (0) |
| B19 5 | negative | negative | negative | negative | negative  (0) |
| B19 6 | negative | negative | negative | negative | negative  (0) |
| B19 7 | negative | negative | negative | negative | negative  (0) |
| B19 8 | negative | negative | negative | negative | negative  (0) |
| B19 9 | negative | negative | negative | negative | negative  (0) |
| B19 10 | negative | negative | negative | negative | negative  (0) |
| B19 11 | negative | negative | negative | negative | negative  (0) |
| B19 12 | negative | negative | negative | negative | negative  (0) |
| B19 13 | negative | negative | negative | negative | negative  (0) |
| B19 14 | negative | negative | negative | negative | negative  (0) |
| B19 15 | negative | negative | negative | negative | negative  (0) |
| B19 16 | negative | negative | negative | negative | negative  (0) |
| B19 17 | negative | negative | negative | negative | negative  (0) |
| B19 18 | negative | negative | negative | negative | negative  (0) |
| B19 19 | negative | negative | negative | negative | negative  (0) |
| B19 20 | negative | negative | negative | negative | negative  (0) |
| B19 21 | negative | negative | negative | negative | negative  (0) |
| DENV1 | negative | negative | negative | negative | negative  (0) |
| DENV2 | negative | negative | negative | negative | negative  (0) |
| DENV3 | negative | negative | negative | negative | negative  (0) |
| DENV4 | negative | negative | negative | negative | negative  (0) |
| DENV5 | negative | negative | negative | negative | negative  (0) |
| DENV6 | negative | negative | negative | negative | negative  (0) |
| DENV7 | negative | negative | negative | negative | negative  (0) |
| DENV8 | negative | negative | negative | negative | negative  (43.41*) |
| DENV9 | negative | negative | negative | negative | negative  (0) |
| DENV10 | negative | negative | negative | negative | negative  (0) |
| DENV11 | negative | negative | negative | negative | negative  (0) |
| DENV12 | negative | negative | negative | negative | negative  (0) |
| DENV13 | negative | negative | negative | negative | negative  (42.37*)_ |
| DENV14 | negative | negative | negative | negative | negative  (0) |
| DENV15 | negative | negative | negative | negative | negative  (0) |
| DENV16 | negative | negative | negative | negative | negative  (38.59*) |
| EBV15 | negative | negative | negative | negative | negative  (0) |
| EBV16 | negative | negative | negative | negative | negative  (0) |
| EBV17 | negative | negative | negative | negative | negative  (0) |
| EBV18 | negative | negative | negative | negative | negative  (0) |
| EBV19 | negative | negative | negative | negative | negative  (0) |
| EBV20 | negative | negative | negative | negative | negative  (0) |
| EBV21 | negative | negative | negative | negative | negative  (0) |
| *above cutoff | | | | | |


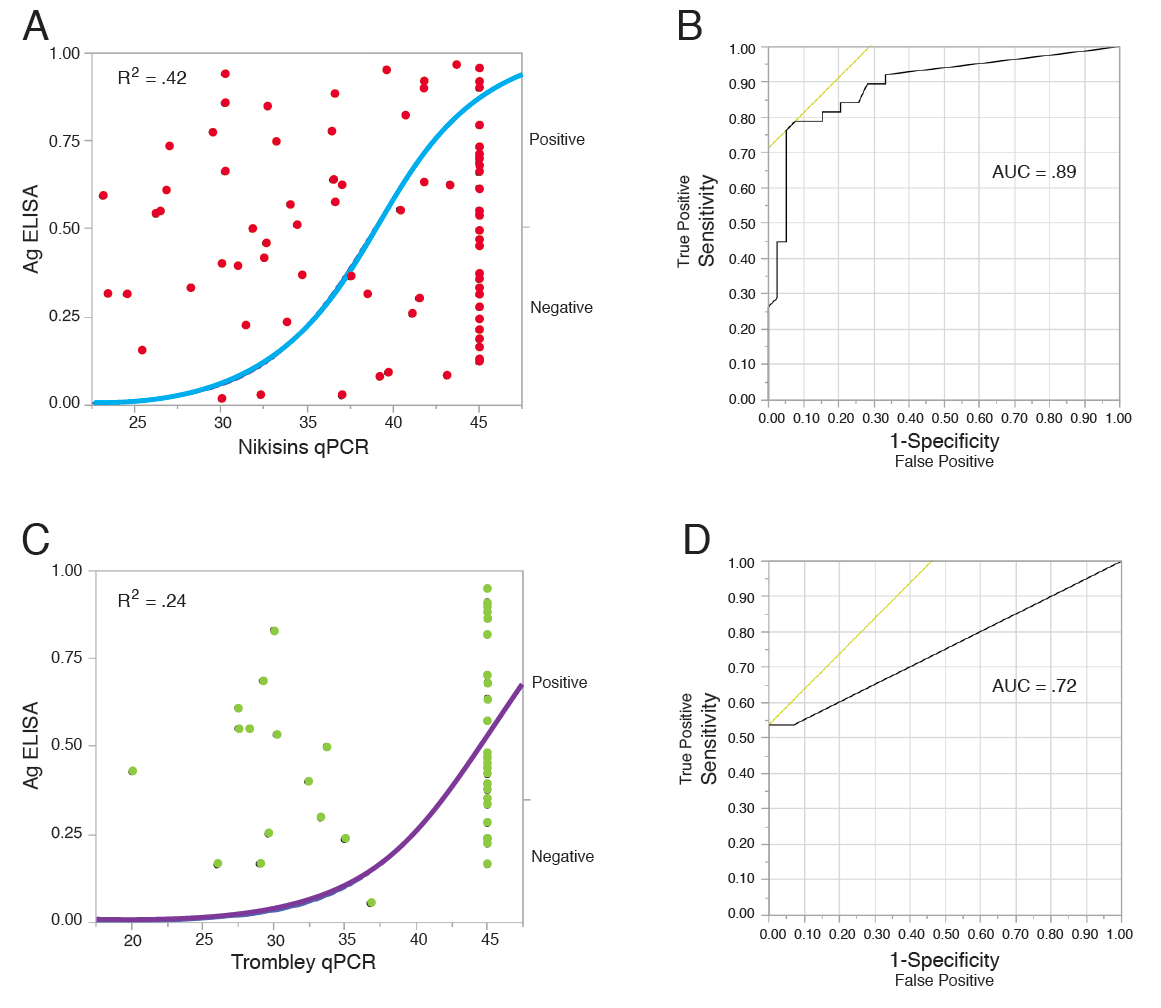


Figure S1. Cut-off determinations for the Nikisins and Trombley and quantitative polymerase chain reaction assays.
